# Inflammation associated anemia and ferritin as disease markers in SLE

**DOI:** 10.1186/ar4012

**Published:** 2012-08-07

**Authors:** Kamala Vanarsa, Yujin Ye, Jie Han, Chun Xie, Chandra Mohan, Tianfu Wu

**Affiliations:** 1 Division of Rheumatic Diseases, Department of Internal Medicine, University of Texas Southwestern Medical School, Dallas, TX 75235, USA

## Abstract

**Introduction:**

In a recent screening to detect biomarkers in systemic lupus erythematosus (SLE), expression of the iron storage protein, ferritin, was increased. Given that proteins that regulate the storage, transfer and release of iron play an important role in inflammation, this study aims to determine the serum and urine levels of ferritin and of the iron transfer protein, transferrin, in lupus patients and to correlate these levels with disease activity, inflammatory cytokine levels and markers of anemia.

**Methods:**

A protein array was utilized to measure ferritin expression in the urine and serum of SLE patients and healthy controls. To confirm these results as well as the role of the iron transfer pathway in SLE, ELISAs were performed to measure ferritin and transferrin levels in inactive or active SLE patients and healthy controls. The relationship between ferritin/transferrin levels and inflammatory markers and anemia was next analyzed.

**Results:**

Protein array results showed elevated ferritin levels in the serum and urine of lupus patients as compared to controls, which were further validated by ELISA. Increased ferritin levels correlated with measures of disease activity and anemia as well as inflammatory cytokine titers. Though active SLE patients had elevated urine transferrin, serum transferrin was reduced.

**Conclusion:**

Urine ferritin and transferrin levels are elevated significantly in SLE patients and correlate with disease activity, bolstering previous reports. Most importantly, these changes correlated with the inflammatory state of the patients and anemia of chronic disease. Taken together, altered iron handling, inflammation and anemia of chronic disease constitute an ominous triad in SLE.

## Introduction

In an effort to identify novel markers of disease activity in systemic lupus erythematosus (SLE), we recently compared serum and urine samples from SLE patients against that of healthy controls, using glass-slide array-based screens. One of the several molecules that were elevated in SLE was ferritin. Given the previous implication of ferritin and other iron-binding proteins (that is, transferrin and hepcidin) in SLE, we pursued this increase further to gauge its clinical significance and possible mechanistic origins.

Ferritin is an acute-phase reactant and is thus elevated in inflammation, autoimmune disorders, chronic infection and liver disease. Elevated levels of ferritin are well established in adult onset Still's disease (AOSD) [1], multiple sclerosis (MS) [2], and in both synovial fluid [3] and synovial cells [4] of rheumatoid arthritis (RA) patients. In RA patients, association between elevated levels of serum ferritin and disease activity score (DAS 28) has previously been reported [5]. High concentrations of serum ferritin levels have been reported in patients with active SLE as compared to inactive SLE [6-8]. Elevated levels of urinary ferritin have also been reported in lupus nephritis (LN) patients [9].

Beyond its role as an acute phase protein, ferritin plays an important role in iron storage and recycling. Ferritin stores iron in a non-toxic and soluble form and releases it in a controlled fashion. Ferritin consists of a unique nanocage structure that resembles a spherical virus and can store up to 4,500 Fe (III) atoms. Ferritin without the associated iron is known as apo-ferritin. Apo-ferritin consists of 24 polypeptide chains of two subunits each: a heavy sub unit (H) and a light subunit (L). The H subunit is involved in iron transport, while the L subunit is responsible for long-term storage of iron in the liver and spleen [10]. In addition to iron storage, another important function of ferritin in humans is its role in macrophages where it recycles iron from old red blood cells (RBCs) and transfers it to apo-ferritin. The iron in transferrin is delivered to immature red blood cells in the bone marrow, thus completing the cycle. Maintaining the balance of iron is crucial at the organism level, and the main function of ferritin is in iron homeostasis and storage of intracellular labile iron [11]. It has been reported that homozygous murine knockouts of ferritin heavy chain are lethal [12].

Ferritin also plays an important role in host immune response as is evident from its increased concentration during infection in order to counter infective agents that attempt to bind iron from the host tissue [13]. An increased immune response augments the migration of ferritin from the plasma to within the cells, so that iron is not available to the infective agent. Two key factors that can regulate ferritin expression are iron [14] and pro-inflammatory cytokines [15,16]. In this study, we assesed the clinical relevance of elevated iron-binding proteins in SLE, and explored their molecular associations.

## Materials and methods

### Patients

Patients were recruited from the renal clinic at Parkland Hospital, an affiliated hospital of the University of Texas Southwestern Medical Center at Dallas. All patient-related procedures were performed strictly following the institutionally approved IRB protocol (UT Southwestern, #STU 082010-119). All patient informed consents were obtained prior to sample collection. Five SLE patients were used for a pilot study using the screening protein array, as described below in the section on protein array. Validation studies were performed using serum and urine samples from an independent cohort of SLE patients using an orthogonal method. All lupus patients fulfill the American College of Rheumatology (ACR) criteria for the diagnosis of SLE. Serum samples were collected from 28 SLE patients. Urine samples were collected from 27 SLE patients, of whom 16 provided both urine and serum. Detailed clinical information is summarized in Table 1.

**Table 1:** Demographics and clinical characteristics of patients used for the serum and urine biomarker validation studies

	Serum^1^	Urine^1^
No.	28	27
Female, no. (%)	25 (89.3)	21 (77.8)
Age, mean +/- SE, years	37.3 ± 1.8	34.6 ± 2.3
Race, African American/Hispanic/Caucasian, no.	17/8/2	13/12/1
SLEDAI, median (interquartile)	10 (3 to 16)	10 (2 to 16)
Renal SLEDAI, median (interquartile)	5 (0 to 8)	5 (0 to 8)
No. of patients with renal SLEDAI = 0 (%)	10 (35.7)	9 (33.3)
Protein:creatinine ratio, mg/mg, mean +/- SE	2.0 ± 0.5	2.3 ± 0.5
Serum Cr, mg/dl, mean +/- SE	1.3 ± 0.2	1.4 ± 0.2
Comorbidities, no. (%)		
Diabetes Melitus	3 (10.7)	4 (14.8)
Hypertension	20 (71.4)	20 (74.1)
Dyslipidemia	12 (42.8)	15 (55.6)
Cardiovascular disease	4 (14.3)	3 (11.1)
Anemia	16 (57.1)	18 (66.7)
Antiphospholipid syndrome	3 (10.7)	3 (11.1)
Venous thromboembolism	3 (10.7)	3 (11.1)
Others	14 (50%)	11 (40.7)
Current medications, no. (%)		
Prednisone	17 (60.7)	20 (74)
Mycophenolic acid	7 (25)	7 (25.9)
Cyclophosphamide	1 (3.6)	2 (7.4)
Azathioprine/MTX	6 (21.4)	3 (11.1)
Cyclosporine/Tacrolimus	2 (7.1)	1 (3.7)
Hydrochloroquine	12 (42.9)	11 (40.7)
Angiotensin blocking agents	14 (50)	16 (59.3)

^1^Note that 16 SLE patients had provided both serum and urine samples.

### Protein array

The serum samples used for the initial array-based screen were from three healthy individuals (mean age, 52; three females, 2AA + 1H) and five patients with SLE (mean age, 33.4; five females, 3AA + 2H; three with active renal disease with SLE disease activity index (SLEDAI) ≥ 10 and renal-related SLE disease activity index (rSLEDAI) ≥ 8). The urine samples used for the initial array-based screen were from three healthy individuals (mean age, 35; three females), and five patients with LN (mean age, 38.3; four females + one male; mean SLEDAI = 19.4; mean rSLEDAI = 9.6). These serum and urine samples were hybridized to glass-slide arrays that interrogate the levels of 274 different human proteins (Raybiotech, Norcross, GA, USA).

### Validation assay

Serum and urine samples obtained from the renal clinic at Parkland Hospital were aliquoted prior to storage at -80°C, and only one aliquot was retrieved for each assay to avoid multiple freeze/thaw cycles. Urine and serum ferritin were measured using a precoated human ferritin ELISA kit from Raybiotech, Inc. Urine and serum transferrin were measured using a human transferrin ELISA kit from Genway Biotech (San Diego, CA, USA). Urine creatinine was measured using a creatinine assay kit from Cayman Chemical (Ann Arbor, MI, USA). Urinary ferritin and urinary transferrin levels were normalized against urine creatinine. Urine and serum cytokines, such as IL-1α, IL-6 and TNF-α, were measured using a Bio-Plex Pro™ Assay kit (Bio-Rad, Hercules, CA, USA), following the manufacturer's instructions.

### Statistics

Data were plotted and analyzed using Graphpad Prism 5 (GraphPad, San Diego, CA, USA). A T-test was used where the normality test passed; otherwise, the nonparametric Mann-Whitney test was used to analyze the data. Likewise, the Pearson method or the nonparametric Spearman method was used for correlation analysis.

## Results

### Increased ferritin levels in SLE

**Figure 1:**
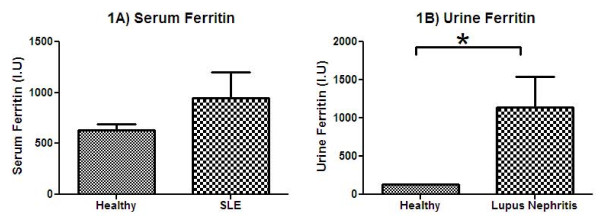
**Serum and urine ferritin levels are increased in patients with lupus**. Ferritin levels in serum and urine of healthy (*N *= 3), SLE (*N *= 5) and LN (*N *= 5) patients were determined using a protein array (Raybiotech, Norcross, GA, USA). Serum ferritin levels were increased by 2.5-fold in SLE patients as compared to healthy controls, and the expression was 18.7-fold higher in the urine of LN patients compared to healthy controls. An unpaired Student's *t***-**test with Welch's correction was applied to compare the means between groups. Data represent the mean ± standard error of mean (SEM). * represents *P *= 0.02.

In an effort to identify novel biomarkers in SLE, sera and urine samples from SLE patients were compared to that of healthy controls using arrays that interrogate 274 different analytes. One of the molecules that was up-regulated in SLE was ferritin (Figure 1). While the increased ferritin levels in SLE patient sera (*N *= 5) measured by this array were not statistically significant (*P *= 0.155), urine ferritin levels were significantly increased (*P *= 0.020) in LN patients compared to healthy controls.

**Figure 2:**
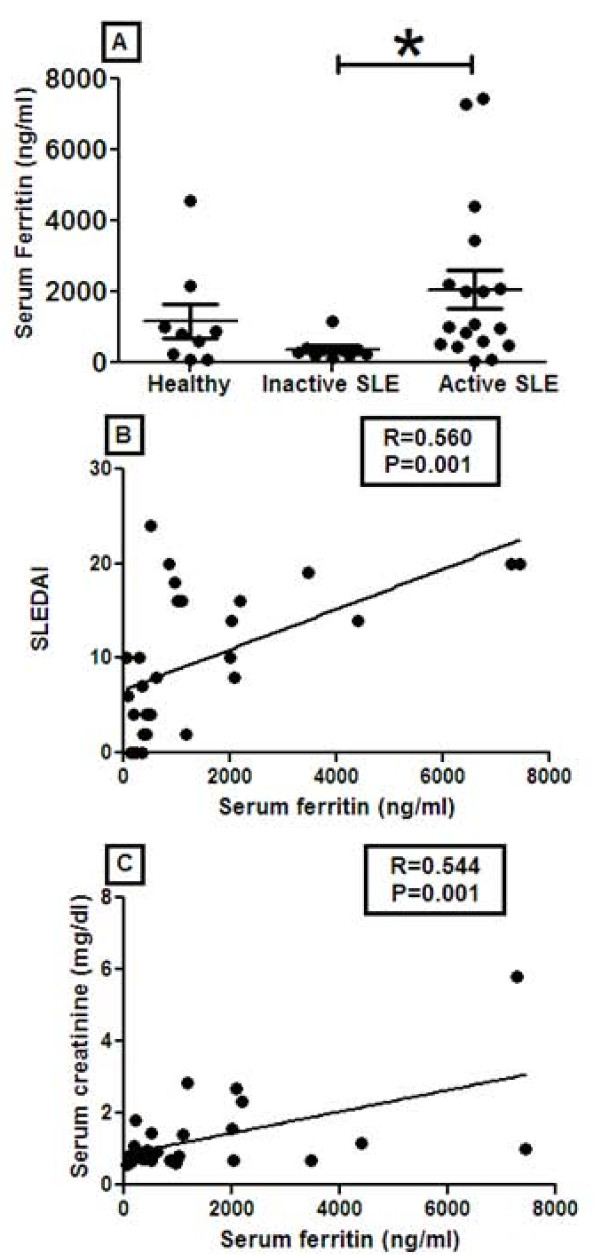
**ELISA analysis of serum ferritin and its correlation with disease activity and serum creatinine levels**. Ferritin levels in serum of healthy (*N *= 9), inactive SLE (*N *= 10), and active SLE (*N *= 18) patients were determined by ELISA (Raybiotech, Norcross, GA, USA). Serum ferritin levels were increased 6.3-fold in active SLE patients compared to healthy controls and 9.8-fold higher in active SLE patients (*P *= 0.014) when compared with inactive SLE patients. Serum ferritin levels correlated significantly with SLEDAI scores (R = 0.56, *P *= 0.001) and serum creatinine levels (R = 0.54, *P *= 0.001).

Next, to validate the array results, serum samples from healthy, inactive SLE and active SLE patients were examined for ferritin levels using ELISA. Ferritin levels were increased significantly (*P *= 0.013) in the sera of active SLE patients as compared to the inactive SLE group. Although SLE patients exhibited elevated serum ferritin (median = 1,058) compared to healthy controls (median = 825), these differences were not statistically significant, possibly due to the raised levels of serum ferritin in one healthy individual (Figure 2).

To determine if increased ferritin expression was related to increased disease activity, serum ferritin titers in SLE patients were compared against SLEDAI scores, serum creatinine concentration and various urine analysis parameters. Serum ferritin levels correlated significantly with SLEDAI (R = 0.56, *P *= 0.001), serum creatinine (R = 0.54, *P *= 0.001), and urine RBC count (R = 0.58, *P *< 0.001) (Figure 2).

**Figure 3:**
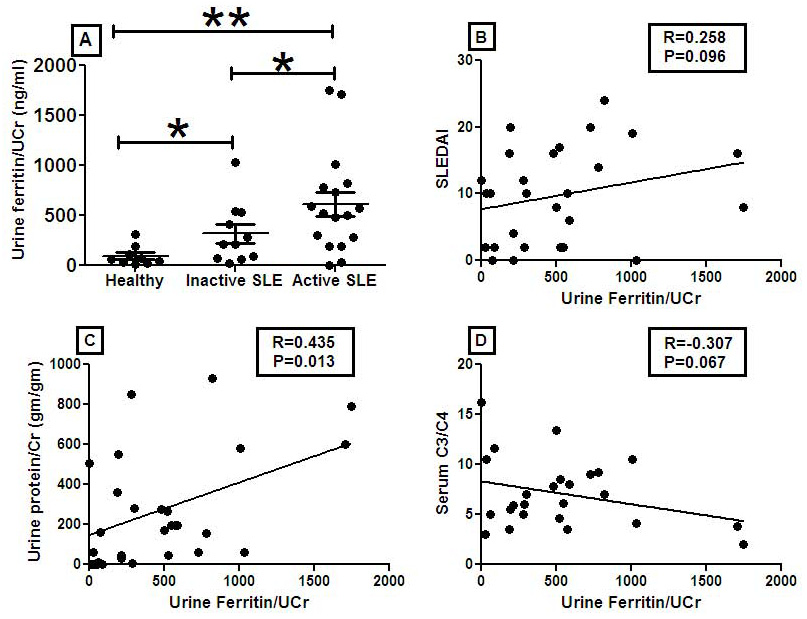
**ELISA analysis of urine ferritin and its correlation with disease activity, urine protein/Cr levels, and complement levels**. Ferritin levels in urine of healthy (*N *= 9), inactive SLE (*N *= 10), and active SLE (*N *= 17) patients were determined by ELISA (Raybiotech, Norcross, GA, USA). Expression of ferritin in the urine of active SLE patients were increased 12-fold as compared to healthy controls (*P *< 0.001), and by 3-fold as compared to inactive SLE patients (*P *= 0.02). Ferritin expression was 4-fold higher in inactive SLE patients as compared to healthy controls (*P *= 0.03). Data represent the mean ± SEM. Urine ferritin/Cr levels correlated significantly with urine protein/Cr levels (R = 0.43, *P *= 0.013), with SLEDAI (R = 0.25, *P *= 0.096), and with serum C3/C4 levels (R = 0.301, *P *= 0.067).

To validate the array findings with the urine samples, urine from healthy, inactive SLE and active SLE patients were examined for ferritin levels by ELISA. Ferritin levels were significantly increased in active (*P *< 0.001) and inactive SLE patients (*P *= 0.03) when compared with healthy controls. There was a significant increase in ferritin levels in the active SLE patients as compared to those with inactive SLE (*P *= 0.02). To determine if increased ferritin expression was related to increased disease severity, urine ferritin levels were next compared against various clinical parameters. Urine ferritin/creatinine (Cr) levels correlated with urine protein/Cr levels (R = 0.43, *P *= 0.013), SLEDAI (R = 0.26, *P *= 0.096), and serum complement (C3/C4) (R = 0.31, *P *= 0.067) (Figure 3).

### Increased ferritin expression in SLE correlates with increased transferrin expression

**Figure 4:**
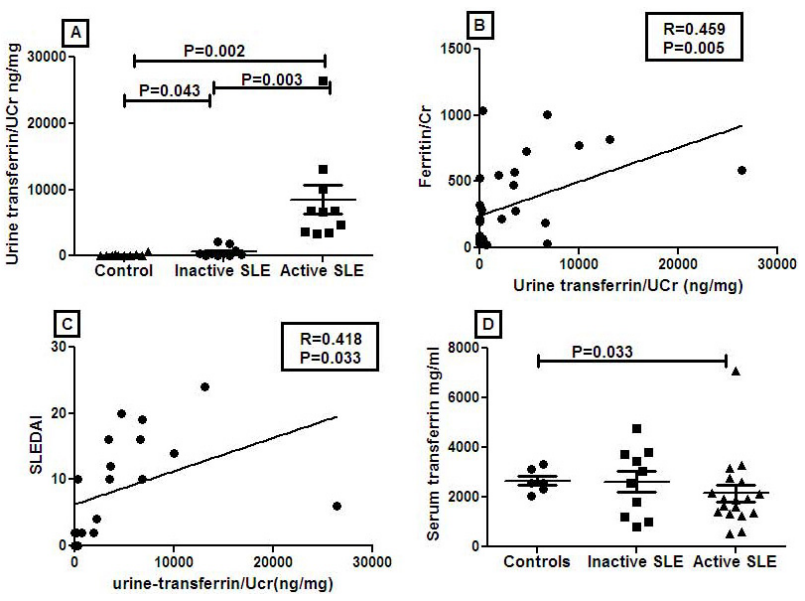
**ELISA analysis of urine and serum transferrin levels**. Transferrin levels in urine of healthy controls (*N *= 10), inactive SLE (*N *= 10) and active SLE (*N *= 10) patients were measured by ELISA (Genway Biotech, San Diego, CA, USA). Transferrin expression was increased by 87-fold (*P *= 0.002) in active SLE patients compared with healthy controls, and by 14-fold (*P *= 0.003) when compared with inactive SLE patients. Urine transferrin/UCr levels correlated significantly with urine ferritin/Cr levels (R = 0.45, *P *= 0.005), disease activity, (R = 0.41, *P *= 0.033), and SLEDAI (R = 0.418, *P *= 0.033). Data represent mean ± SEM.

Given that transferrin is another iron-binding protein that has been reported to be altered in SLE [17], we asked if these patients also exhibited elevated transferrin levels. Serum and urine samples of active and inactive SLE patients and healthy controls were analyzed for transferrin levels by ELISA. Urine transferrin levels were significantly increased in active and inactive SLE patients when compared with healthy controls. Also, transferrin levels were raised in inactive SLE patients when compared with controls. Elevated urine transferrin/Cr levels correlated significantly with urine ferritin levels (R = 0.46, *P *= 0.005), SLEDAI scores (R = 0.42, *P *= 0.033), and rSLEDAI (R = 0.49, *P *= 0.013) (Figure 4). In contrast to urine transferrin, serum transferrin levels were in fact lower in active SLE patients (Figure 4D).

### Correlation of elevated ferritin levels with inflammation and anemia in SLE

**Figure 5:**
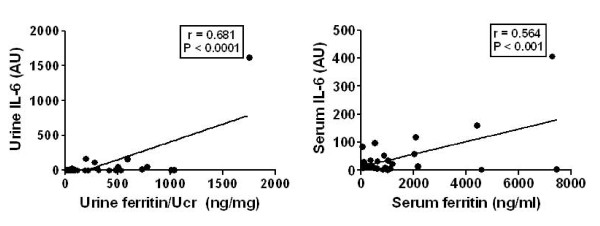
**Correlation of serum and urine ferritin levels with inflammatory cytokine levels**. Serum and urine ferritin levels determined using ELISA were correlated with inflammatory cytokine levels. Urine ferritin levels correlated with urine IL-6 (r = 0.68, *P *< 0.001) and serum IL-6 (r = 0.56, *P *< 0.0001).

**Figure 6:**
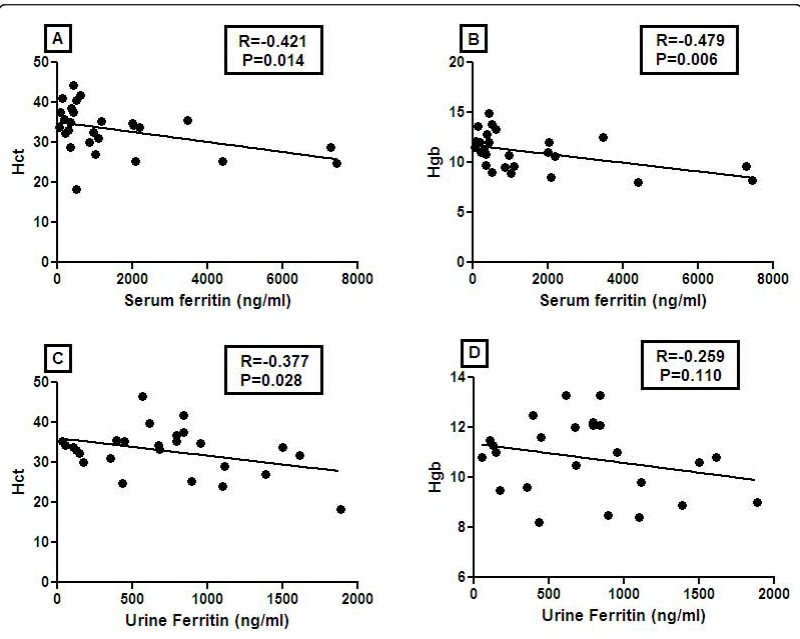
**Correlation of serum and urine ferritin with hematocrit (Hct) and hemoglobin (Hgb)**. Serum and urine ferritin levels measured by ELISA were correlated with Hct and Hgb. Serum ferritin correlated negatively with Hct (R = -0.42, *P *= 0.012) and Hgb (R = -0.47, *P *= 0.006). Urine ferritin levels also correlated negatively with Hct (R = -0.37, *P *= 0.028) and Hgb (R = -0.25, *P *= 0.110), although the correlation with Hgb was comparatively weaker.

In light of the previous reports that inflammatory cytokines may regulate ferritin expression [15,16], we further examined whether the patients with elevated ferritin levels also had elevated levels of inflammatory cytokines, using the data from the original array-based screening. Serum and urine ferritin levels of healthy (*N *= 3), SLE (*N *= 5), and LN patients (*N *= 5), as measured by the protein arrays, were analyzed for possible correlation with serum levels of interleukin (IL)-1α, IL-6, and tumor necrosis factor (TNF)α, also interrogated on the same arrays. Urine ferritin levels correlated significantly with urine IL-6 (*P *< 0.001, Figure 5 and Additional file 1), and with TNF-α (*P *= 0.02, Additional file 1, and IL-1α (*P *= 0.07, Additional file 1). Although the serum ferritin levels exhibited similar trends, these correlations did not attain statistical significance. Interestingly, serum and urine ferritin levels in SLE patients also correlated with anemia, as marked by the reduced hemoglobin and hematocrit (Figure 6).

## Discussion

The present study reveals urinary elevations in two key iron-binding proteins (ferritin and transferrin) in SLE patients, the levels of which were found to be correlated with each other, as well as with disease activity, inflammatory status and anemia. Collectively, this constellation of abnormalities may serve as a marker of disease severity in SLE.

Previously, Shoenfeld and coworkers provided evidence for hyperferritinemia in lupus [18,19], where it was suggested to be a biomarker in lupus. Specifically, hyperferritinemia was associated with thrombocytopenia, lupus anticoagulant and anti-cardiolipin antibodies, suggesting that it may be an early marker of antiphospholipid syndrome in SLE [18]. Likewise, in a Japanese population, Nishiya *et al. *demonstrated elevated levels of serum ferritin in SLE patients as compared to RA patients and also reported that these increased levels positively correlated with SLEDAI and negatively with complement levels [6]. Another study by Beyan *et al. *showed a similar rise in serum ferritin levels in Turkish SLE patients and a significant change in ferritin levels before and after treatment, and also showed a positive correlation between serum ferritin levels and SLEDAI [8]. A study of a Korean cluster of SLE patients by Lim *et al. *also suggested similar results and indicated the use of ferritin as a marker of disease activity in SLE patients [7]. However, these studies did not relate the change in ferritin levels to inflammation or anemia. The present study is the first of its kind to be conducted in African American, Hispanic and Caucasian SLE patients. Our results demonstrate a significantly positive and linear correlation of serum ferritin levels with SLEDAI, serum creatinine levels and urine RBC counts, and a weaker correlation with serum complement. This study, along with previous reports by other groups, indicates that if the basal levels of ferritin in SLE patients are known, a significant variation in these levels can be used as a reliable indicator of disease activity. Given recent studies showing that increased serum ferritin levels are associated with increased risk of atherosclerotic coronary artery disease (CAD) and myocardial infarction [20], serum ferritin may also be useful in tracking CAD, a leading cause of morbidity and mortality in SLE.

As has been previously reported for urinary ferritin levels in LN [9], our study demonstrates a significant increase in urinary ferritin levels (normalized against urinary creatinine) in active SLE, as detected using an ELISA- and protein array-based approach. Furthermore, urinary ferritin levels showed a positive correlation with urinary protein/Cr levels, and a negative correlation with levels of serum complement (C3/C4).

Ferritin synthesis is regulated by intracellular iron at both the transcriptional and translational levels. When iron levels are low, ferritin synthesis is reduced and vice versa. Ferritin regulation by iron is largely posttranscriptional [14]. The 5' end of the heavy and light chain ferritin mRNA contains an iron responsive element (IRE) that has a stem-loop structure. RNA-binding proteins, known as iron regulatory proteins IRP1 and IRP2, bind to this stem loop structure and inhibit the translation of ferritin mRNA, with different tissue-specific roles [21-23].

The positive correlation between serum ferritin levels and proinflammatory cytokines observed in our study is supported by a number of reports in the literature. The proinflammatory cytokines, TNFα and IL-1α, are known to stimulate the expression of ferritin by inducing the transcription of the H ferritin gene in mouse adipocytes and human muscle cells [15]. Also, translation of ferritin is induced by IL-6, TNFα and IL-1α in the HepG2 hepatic cell line [16], and iron is essential for this regulation as the fact that the upregulation of ferritin is inhibited by deferoxamine [24]. Cytokines indirectly regulate ferritin translation by inducing synthesis of nitric oxide synthase, which in turn increases nitric oxide (NO), an activator of both IRP1 and IRP2 [25]. Lipopolysaccharides (LPS), a component of the outer membrane of gram-negative bacteria elicit a number of reactions that involve ferritin. Although LPS is known to stimulate a number of inflammatory cytokines, there are reports where endotracheal administration of LPS in rats increased ferritin mRNA expression [26]. Ferritin is also regulated by oxidative stress directly by targeting specific genes or indirectly by the modification of IRPs.

In mouse models, the macrophage is the main source of serum ferritin [27]. During immune responses, activated macrophages produce more ferritin, which results in elevated serum ferritin in the circulation. Human CD4+ and CD8+ T lymphocytes and CD19+ B lymphocytes express H-ferritin binding sites, which are positively associated with the proliferative status of these immune cells [28]. In ferritin treated mice, clonal expansion and maturation of precursor T cells into effector T cells was suppressed, suggesting that ferritin has immunosuppressive effects on lymphocyte proliferation [29]. Both L- and H-rich ferritin exert their inhibitory activity on immunoglobulin generation by B cells in a T-independent as well as T-dependent way [28]. The immunosuppressive effects of ferritin may be mediated by IL-10 production from regulatory T cells [30]. Patients with rheumatoid arthritis (RA) have higher concentrations of free iron and lactoferrin, the latter may play a role in preventing toxic damage to the synovium from free iron which accumulates during the inflammatory response [31].

Taken together with the above mechanistic studies, our findings suggest that inflammation in SLE is likely to be a major driving force in up-regulating ferritin expression. Inflammation is also well established to up-regulate hepcidin-mediated degradation of the iron transport channel, ferroportin, thereby sequestering iron in the form of ferritin in the cells of reticulo endothelial system. This results in the unavailability of iron for Hb production, hence explaining the anemia. A further feature of "anemia of chronic inflammation" is raised ferritin coupled with reduced transferrin, a scenario witnessed in our present study.

It is instructive to reconsider the salient differences between iron deficiency anemia and anemia of chronic disease. Iron-deficiency anemia and 'anemia of chronic disease' both exhibit reduced serum iron but are very different otherwise. Whereas anemia of chronic disease is marked by elevated ferritin but reduced transferrin (and total iron-binding capacity (TIBC)) levels, iron deficiency anemia exhibits the reverse profile. Indeed, the SLE patients described in this report exhibit 'anemia of chronic disease' rather than iron deficiency anemia. One could certainly further demonstrate this by examining bone marrow iron stores (increased in chronic inflammation but depleted in iron-deficiency anemia), but this is beyond the scope of this report. Given that most SLE patients with anemia have anemia of chronic disease rather than iron-deficiency anemia, it is important to manage this accordingly in the clinics. Targeting the underlying inflammation rather than treating it with iron supplements may be a more fruitful approach in these patients.

There are several caveats of note in the present study. As discussed above, examination of iron stores in the bone marrow could further substantiate the presence of "anemia of chronic disease" in SLE. Since the current studies are cross-sectional in nature, we do not yet know if the markers described in this report truly have disease-predictive value. Future longitudinal studies are clearly warranted to test the predictive potential of these markers in SLE. In addition, further studies will be needed to understand the impact of medications on ferritin levels, and the impact of ferritin on the ongoing autoimmune process. Finally, one needs to explore if anemia of chronic disease is also a prominent feature of other chronic diatheses, including RA.

In conclusion, we have established a positive linear correlation between ferritin levels in SLE and disease activity, thus bolstering previous reports in other ethnic groups. Most importantly, the changes in ferritin and transferrin levels correlated with the inflammatory state and anemia in SLE, making them potential markers of disease activity. Taken together, inflammation → elevated ferritin → "anemia of chronic inflammation" may constitute an ominous triad in SLE.

## Conclusions

Urinary ferritin and transferrin levels are increased significantly in SLE patients and correlated with disease activity, supporting previous reports in other ethnic groups. Most importantly, the elevation of ferritin and transferrin correlated with the inflammatory state and anemia of chronic disease suggesting that hyperferritinemia could potentially play a role in regulating immunity. Taken together, altered iron handling, inflammation and anemia of chronic disease may constitute an ominous triad in SLE.

## Abbreviations

ACR: American College of Rheumatology; AOSD: adult onset Still's disease; CAD: coronary artery disease; Cr: creatinine; DAS 28: Disease Activity Score 28; IRE: iron responsive element; LN: lupus nephritis; LPS: lipopolysaccharide; MS: multiple sclerosis; NO: nitric oxide; RA: rheumatoid arthritis; rSLEDAI: renal-related SLE disease activity index; SLE: systemic lupus erythematosus; SLEDAI: SLE disease activity index; TIBC: total iron-binding capacity; TNF: tumor necrosis factor.

## Competing interests

The authors declare that they have no competing interests.

## Authors' contributions

TW and CM conceived of the study, designed the study and drafted the manuscript. KV, YY, JH and TW carried out the screening and validation studies, and performed the statistical analysis. All authors read and approved the final manuscript.

## Supplementary Material

Additional file 1 **Figure S1. Correlation of serum and urine ferritin levels with inflammatory cytokine levels**. Serum and urine ferritin levels determined by using a protein array were correlated with cytokine levels measured in the same assay. Urine ferritin levels correlated with IL-6 (R = 0.94, *P *< 0.001), TNF α (R = 0.71, *P *= 0.022), and IL-1α *(P = 0.073)*. Serum ferritin levels showed weaker correlations with proinflammatory cytokine levels. Click here for file
