# Supplementary figures and images for: Inflammation associated anemia and ferritin as disease markers in SLE
Source: Arthritis Res Ther. 2012 Aug 7;14(4):R182. doi: 10.1186/ar4012 (PMC3580577; doi:10.1186/ar4012)

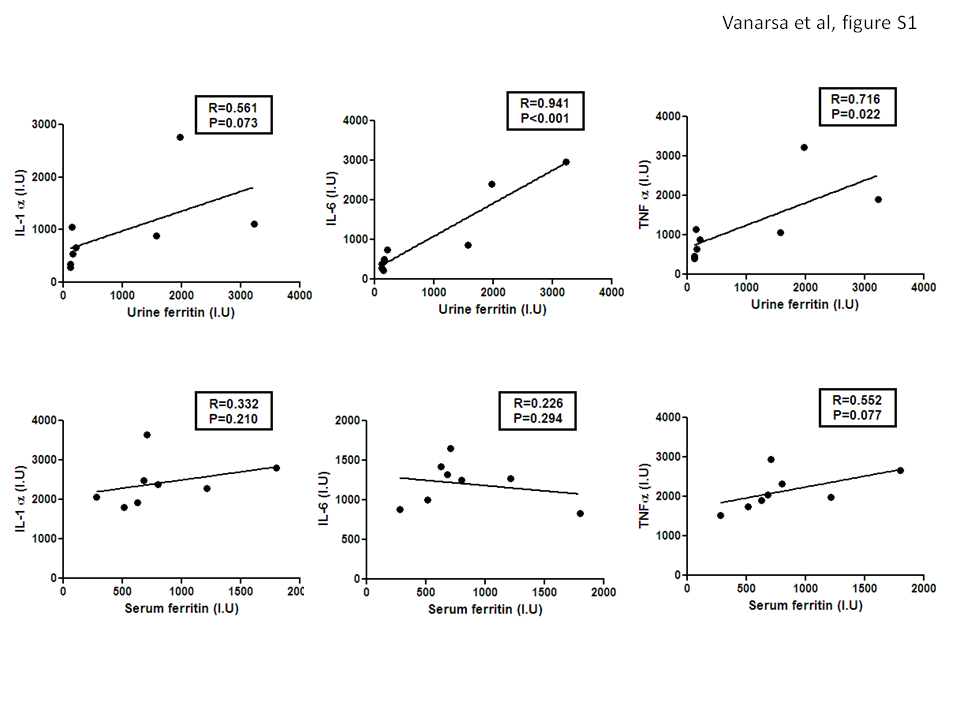

Supplement: Additional file 1 — Figure S1. Correlation of serum and urine ferritin levels with inflammatory cytokine levels. Serum and urine ferritin levels determined by using a protein array were correlated with cytokine levels measured in the same assay. Urine ferritin levels correlated with IL-6 (R = 0.94, P < 0.001), TNF α (R = 0.71, P = 0.022), and IL-1α (P = 0.073). Serum ferritin levels showed weaker correlations with proinflammatory cytokine levels. [file ar4012-S1.TIFF]
